# The Ghrelin Signalling System Is Involved in the Consumption of Sweets

**DOI:** 10.1371/journal.pone.0018170

**Published:** 2011-03-23

**Authors:** Sara Landgren, Jeffrey A. Simms, Dag S. Thelle, Elisabeth Strandhagen, Selena E. Bartlett, Jörgen A. Engel, Elisabet Jerlhag

**Affiliations:** 1 Department of Pharmacology, The Sahlgrenska Academy, University of Gothenburg, Gothenburg, Sweden; 2 Ernest Gallo Clinic and Research Center, University of California San Francisco, Emeryville, California, United States of America; 3 Department of Biostatistics, Institute of Basic Medical Science, University of Oslo, Oslo, Norway; 4 School of Public Health and Community Medicine, The Sahlgrenska Academy, University of Gothenburg, Gothenburg, Sweden; National Institute on Aging Intramural Research Program, United States of America

## Abstract

The gastric-derived orexigenic peptide ghrelin affects brain circuits involved in energy balance as well as in reward. Indeed, ghrelin activates an important reward circuit involved in natural- as well as drug-induced reward, the cholinergic-dopaminergic reward link. It has been hypothesized that there is a common reward mechanism for alcohol and sweet substances in both animals and humans. Alcohol dependent individuals have higher craving for sweets than do healthy controls and the hedonic response to sweet taste may, at least in part, depend on genetic factors. Rat selectively bred for high sucrose intake have higher alcohol consumption than non-sucrose preferring rats and vice versa. In the present study a group of alcohol-consuming individuals selected from a population cohort was investigated for genetic variants of the ghrelin signalling system in relation to both their alcohol and sucrose consumption. Moreover, the effects of GHS-R1A antagonism on voluntary sucrose- intake and operant self-administration, as well as saccharin intake were investigated in preclinical studies using rodents. The effects of peripheral grelin administration on sucrose intake were also examined. Here we found associations with the ghrelin gene haplotypes and increased sucrose consumption, and a trend for the same association was seen in the high alcohol consumers. The preclinical data show that a GHS-R1A antagonist reduces the intake and self-administration of sucrose in rats as well as saccharin intake in mice. Further, ghrelin increases the intake of sucrose in rats. Collectively, our data provide a clear indication that the GHS-R1A antagonists reduces and ghrelin increases the intake of rewarding substances and hence, the central ghrelin signalling system provides a novel target for the development of drug strategies to treat addictive behaviours.

## Introduction

Human imaging studies reveal an underlying disruption in the reward systems in addictive behaviors including alcohol use disorder and binge eating [1], [2], [3] and common neurobiological mechanisms may underlie these diseases. Recently, ghrelin and its receptor (GHS-R1A) have been implied for such roles [4], [5], [6]. The gastric-derived orexigenic peptide, ghrelin [7], affects brain circuits involved in energy balance [8] as well as in reward [9], [10]. Indeed, ghrelin activates an important reward circuit involved in natural- as well as drug-induced reward, the cholinergic-dopaminergic reward link [11], [12]. This reward link encompasses a dopaminergic projection from the ventral tegmental area (VTA) to the nucleus accumbens (N.Acc.) that forms part of the mesolimbic dopamine system, together with a cholinergic projection from the laterodorsal tegmental area (LDTg) to the VTA. Ghrelin may, via activation of this reward link, increase the incentive value of motivated behaviors such as food and drug seeking [10]. Supportively, alcohol-, cocaine- as well as amphetamine-induced reward, as measured by locomotor activity, accumbal dopamine release and conditioned place preference, are suppressed by ghrelin receptor (GHS-R1A) antagonism [5], [13]. Further, genetic, pharmacologic and surgical rodent models of altered ghrelin signalling has been used to show that ghrelin action at the level of the VTA is important for the intake of and motivation to obtain palatable/rewarding food [6].

The hypothesis of a common reward mechanism for alcohol and sweet substances also transfers to humans. Interestingly, alcohol dependent individuals have higher craving for sweets than do healthy controls [14]. The hedonic response to sweet taste may, at least in part, depend on genetic factors [15] which have been shown in both humans and animals in numerous studies [16], [17], [18], [19], [20], and is modulated via several mechanisms, which include the mesolimbic dopamine system. Specifically, sweet tasting substances (both caloric and non-caloric) increases the firing of accumbal dopamine [21].

In an attempt to translate the preclinical data regarding ghrelin, sucrose and alcohol into humans, a group of alcohol-consuming individuals selected from a population cohort was investigated for genetic variants of the ghrelin signalling system in relation to both their alcohol and sucrose consumption. Moreover, the effects of GHS-R1A antagonism on voluntary sucrose- intake and operant self-administration, as well as saccharin intake were investigated in preclinical studies using rodents. To exclude the possibility of taste aversion by the GHS-R1A antagonist, such experiments were conducted in mice. Additionally, the role of peripheral ghrelin administration on sucrose consumption in fed rats was examined.

## Materials and Methods

### Ethics statement

All human subjects gave their written informed consent to the study, and the protocol was approved by the local ethics committee in Gothenburg, Forskningsetikkommitté Ö (ethics number Ö 237-00). This study was performed according to the tenets of the Helsinki Declaration.

The experiments with mice were approved by the Ethics Committee for Animal Experiments in Gothenburg, Sweden, and the experiments with Long Evan rats were pre-approved by the Gallo Center Institutional Animal Care and Use Committee and were in accordance with NIH guidelines for the Humane Care and Use of Laboratory Animals (ethics number 80-07 and 09.02.191).

### The population cohort INTERGENE

The individuals of the human genetic study were selected from the population cohort INTERGENE. INTERGENE is a population based research program that assesses the INTERplay between GENEtic susceptibility and environmental factors for the risk of chronic diseases in western Sweden. The study procedure is described in detail elsewhere [22], [23], [24] and at http://www.intergene.gu.se. In the main questionnaire the participants were, among other things, asked about the frequency of intake of different types of alcoholic beverages (low alcohol beer, medium-strong beer, strong beer, wine, dessert wine and spirits) as well as on intake of high-sugar containing food. The data on frequencies and standardized portions of alcoholic beverage consumed per occasion were used to calculate the total consumption of pure alcohol in g/day. For the purpose of the present study low- (n = 296) and high (n = 283) alcohol consuming individuals were selected from the total cohort on the basis of their alcohol consumption (0.3–1.7 g and 20–196 g EtOH/day, respectively). For all these individuals, the total sucrose intake (g sucrose/day) was calculated from the section of the above mentioned questionnaire regarding eating habits including consumption of sweets (e.g. candy, marmalade, jam, cookies, cake and juice). Blood samples for genetic analyses were collected from all individuals. The study sample is more thoroughly described in Table 1.

**Table 1:** Description of study material and studied variables.

Variable		High EtOH consumersn = 283	Low EtOH consumersn = 296	p-value
Age	(years)	55 (13)	57 (14)	0.089
Height	(cm)	177 (8)	168 (9)	<0.001
Weight	(kg)	82 (13)	78 (15)	<0.001
BMI	(kg/m^2^)	26 (3)	27 (5)	0.001
Sugar intake	(g/day)	49 (39)	46 (40)	0.095
Ethanol Intake	(g/day)	30 (16)	1 (0.4)	
Gender	Male	252 (89%)	96 (32%)	<0.001
	Female	31 (11%)	200 (68%)	
Smoking	Yes	193 (69%)	145 (49%)	<0.001

*Data are presented as mean (SD) or as absolute numbers (%) for study variable; p-values are calculated using Mann Whitney U test for continuous, and Chi^2^ test for categorical study variables respectively.*

### Genotyping and genetic association study statistics

All individuals were genotyped for 6 tag SNPs in the pro-ghrelin gene (*GHRL;* rs4684677, rs42451, rs35680, rs34911341, rs696217, rs26802) and 4 tag SNPs of the ghrelin receptor gene (*GHSR,* rs2948694, rs572169, rs2232165, rs495225). Genotyping was performed using TaqMan Pre-Designed SNP Genotyping Assays® (Applied Biosystems, Foster City, CA, USA) on the ABI PRISM 7900HT Sequence Detection System (Applied Biosystems, Foster City, CA, USA) using the TaqMan Allelic Discrimination technology [25]. The tag SNPs used are the same as in our previous studies on the ghrelin system and alcohol dependence [26], [27].

Deviation from Hardy Weinberg equilibrium (HWE) for alleles at individual loci was tested. Differences in clinical characteristics between groups were analyzed by use of Chi^2^-test for categorical variables, and Mann-Whitney *U*-test for continuous variables. Identification of significant covariates for each outcome variable was made using forward stepwise regression models. Single marker associations were performed using regression models including relevant covariates in an additive model (dd = 0, Dd = 1 and DD = 2, where D = minor allele and d = major allele). Haplotype analysis was used to identify the haplotype window with strongest association using forward stepwise logistic or linear haplotype regression (cut-off p = 0.01) always keeping the identified covariates in the model. To this end, haplotype frequencies were estimated using the expectation-maximization algorithm [28] yielding all possible haplotypes present in our study population. In subsequent analyses, however, only haplotypes with an overall estimated frequency of >5.0% were included, while the rarer haplotypes were pooled. The p-value threshold for statistical significance used in this study was p = 0.05. To correct for multiple testing, Bonferroni correction for the number of studied SNPs (n = 10) was used in the single marker analysis and permutation (10 000 permutations) was used in the sliding window analyses. The corrected p-values are designated p_c_. The softwares used for the statistical analyses were SYSTAT11 (SYSTAT Software GmbH, Erkrath, Germany) and HelixTree 6.3 (Golden Helix, Bozeman, MT, USA).

### Animals

Both mice and rats were used for the preclinical experiments. C57BL/6 mice (23–36 g body weight; B&K Universal AB, Sollentuna, Sweden) were used for the saccharin intake and taste aversion experiments. This strain was used since it has been used extensively in the laboratory [5], [29], [30]. Long–Evans rats (Harlan, Indianapolis, IN, USA), were used for the intermittent access 20% sucrose two-bottle-choice drinking paradigm, operant sucrose self-administration procedures as well as the 5% sucrose two-bottle-choice drinking paradigm, since such studies are well-documented in this strain [31], [32], [33]. All animals were given time to acclimatize to the individual housing conditions and handling before the start of the experiments. They were individually housed in ventilated plexiglass cages and were maintained at 20°C with 50% humidity. Animals in the two-bottle choice experiments were maintained on a 12 hour reversed light dark cycle (lights off at 10 am) and Long-Evans rats in the operant self-administration experiments were maintained on a regular 12 hour light-dark cycle (lights on at 7 a.m.). Food and water were available *ad libitum*, except for short periods during initial training in the operant self-administration paradigm, as described below. In all experiments the weight of each animal was measured daily prior to bottle presentation, for calculating the grams of saccharin/sucrose intake per kilogram of body weight (g/kg). All experiments were performed in adult post-pubertal age-matched male rats or mice.

### Drugs

For mice, the selected dose of JMV2959, 6 mg/kg (i.p.), (synthesized at the Institute des Biomolécules Max Mousseron (IBMM), UMR5247, CNRS, Montpellier 1 and 2 Universities, France), a GHS-R1A antagonist, was determined previously [5]. In the saccharin intake and taste aversion experiments this dose did not affect the gross behavior of the mice. For Long-Evans rats the selected doses were 1, 2 and 3 mg/kg JMV2959 since these doses have been found to reduce alcohol intake and operant self-administration in Long-Evans rats [34]. JMV2959 was always administered 20 minutes prior to sucrose/saccharin exposure. It has in previous studies been established as a GHS-R1A antagonist [35]. JMV2959 was dissolved in vehicle (0.9% sodium chloride). A balanced design was used in all drug challenges. The peripheral (i.p.) administration of JMV2959 allowed studying repeated effects over several days. Acylated rat ghrelin (Bionuclear; Bromma, Sweden) was diluted in 0.9% sodium chloride (saline vehicle) and was administrated peripehrally (i.p.) (1 ml/kg body weight). The selected dose, 0.33 mg/kg, was determined previously, as it increases locomotor activity and accumbal dopamine release as well as induces a conditioned place preference in mice [36] and to increase the motivation to consume sucrose in rats [37]. Ghrelin was administered 10 min prior to the initiation of the experiments, i.e. when light was turned out and the sucrose bottle was presented. Lithium chloride (LiCl) (Sigma Ultra, Sigma Chemicals CO; Stockholm, Sweden) was diluted in vehicle (0.9% sodium chloride) and was administered at a dose previously reported to produce aversion (150 mg/kg, i.p.). No gross behavioral effects of LiCl were observed in these experiments. For all experiments 0.9% sodium chloride was used as vehicle.

### Intermittent Access 5% Sucrose Two-bottle-choice Drinking Paradigm

The intermittent access 5% sucrose two-bottle-choice drinking paradigm is very similar to the intermittent access paradigm for alcohol [31]. In brief, rats (n = 12) were given access to one bottle of 5% sucrose and one bottle of water for three 24-hour-sessions per week (Mondays, Wednesdays and Fridays), 15 minutes after the lights went out in a reversed light/dark cycle room. The rats had unlimited access to two bottles of water between the sucrose-access-periods. Bottles were weighed at 30 minutes, 6 hours and 24 hours after the fluids were presented and measurements were taken to the nearest 0.1 gram. The weight of each animal was measured daily prior to bottle presentation, for calculating the grams of sucrose intake per kilogram of body weight (g/kg). The preference for sucrose over water (the ratio of sucrose to total fluid intake) was calculated at all time points. Drug administration began once the animals had attained stable drinking levels of sucrose about 7 weeks (approximately 20 drinking sessions). JMV2959 (1, 2, and 3 mg/kg) or vehicle (saline) were administered 20 minutes before the presentation of the sucrose bottles. Each injection was given 7 days apart using a Latin square design, thus each animal served as its own control. Between the injection days, the rats were exposed to their normal drinking schedule of intermittent access as described above with no injections for the remaining days of that week. Sucrose was diluted in tap water to a final concentration of 5% (w/v). The data were analyzed by repeated-measures ANOVA, followed by a Newman–Keuls post hoc analysis.

### Operant Sucrose Self-Administration

#### Apparatus

Testing was conducted in standard operant conditioning chambers (Coulbourn Instruments, Allentown, PA) enclosed in ventilated, sound-attenuating cubicles. Each chamber housed two retractable levers on the right wall with a liquid dipper system placed centrally between them. A house light was present on the wall opposite the levers and remained on at all times during the operant session. Stimulus lights were present above each lever. An apparatus to emit a tone under specific operant conditions was also present. Upon correct (active) lever press(es), the stimulus light above the active (right) lever was illuminated for 3 s and was accompanied by a 3-s tone to reinforce availability of reward in the dipper receptacle. The dipper port was illuminated for 10 s while the dipper cup was available. Stimulus, fluid delivery, and operant responses were all controlled and recorded by a computer (Coulbourn Instruments) by using Graphic State 2.0 software.

#### Operant sucrose self-administration paradigm

Self-administration testing was conducted in standard operant conditioning chambers (Coulbourn Instruments, Allentown, PA) as described previously [32], [33]. In brief, rats (n = 15) and trained to self-administer 5% sucrose, on a fixed ratio 3 (FR3; three active lever presses required for 0.1 ml reward) schedule of reinforcement, daily (Monday through Friday) for 30 minutes. Rats were kept on the FR3 protocol for at least 20 sessions before treatment. To evaluate the acute effect of JMV2959 on sucrose self-administration, JMV2959 (1, 2, and 3 mg/kg) or vehicle (saline) were administered 20 minutes before the operant session. Each injection was given 7 days apart in a Latin square design, thus each animal served as its own control. Between the injection days, the rats were exposed to their normal schedule of reinforcement as described above with no injections for the remaining days of that week. Additionally, food intake was measured in the 24 hour period following the drug challenge in order to further examine the non-specific appetitive effects of JMV2959. The data were analyzed by repeated-measures ANOVA, followed by a Newman–Keuls post hoc analysis.

### Saccharin consumption paradigm

After one week of habituation the mice were housed individually with continuous access to tap water and saccharin solution (0.1%) for four weeks. Thereafter, the saccharin solution was limited to the first three hours of the dark period and this limited access paradigm was maintained for three weeks prior to GHS-R1A antagonist treatment. Food and water was freely supplied during the entire day. JMV2959 or vehicle was administered for two subsequent days, due to advantages with peripheral administrations. Thereafter, the mice were untreated for two days and the saccharin intake was measured these days as well. These data were analyzed as the average three hour intake over the two treatment days. In all experiments the intake of saccharin and water were measured throughout the three hour drinking session. The 24 hour food intake was also measured. The measurements of saccharin consumption are expressed per gram body weight. The effects of GHS-R1A treatment on intake in mice were evaluated by a two-way ANOVA followed by Bonferroni post hoc test.

### Taste aversion experiment

Food and water were available *ad libitum* during the taste aversion experiments. Saccharin solution (0.1%) was presented the first three hours of the dark period and this procedure was repeated every other day for a total of three saccharin drinking days (Monday, Wednesday and Friday). At the end of the three hours of saccharin consumption JMV2959, LiCl or vehicle was administered. The first day (Monday) is considered as baseline since the drugs were administered after saccharin consumption. No saccharin or drug was presented the days in between treatment (Tuesdays, Thursday and Saturday). The last day (Sunday) saccharin was presented but no drug was administered. The effects of GHS-R1A treatment on intake in mice were evaluated by a two-way ANOVA followed by Bonferroni post hoc test. However, the intake at baseline and after treatment was analyzed with a one-way ANOVA followed by Bonferroni post hoc test.

### Sucrose Consumption Paradigm

The 5% sucrose two-bottle-choice drinking paradigm is adapted from a previous sucrose consumption paradigm [38]. In brief, rats (n = 16) were given access to one bottle of 5% sucrose and one bottle of water for three hours for three subsequent days (Monday, Tuesday, Wednesday) and a baseline intake of fluids and food was established. The following week the rats had access to sucrose bottle and were subjected to peripheral drug administration of either ghrelin (0.33 mg/kg) or vehicle Monday and Wednesday. The rats either received ghrelin (0.33 mg/kg) or vehicle day one and vice versa treatment day two, thus each animal served as its own control. The rats had unlimited access to two bottles of water between the sucrose-access-periods. Bottles were weighed three hours after the fluids were presented and measurements were taken to the nearest 0.1 gram. The weight of each animal was measured daily prior to bottle presentation, for calculating the grams of sucrose intake per kilogram of body weight (g/kg). The preference for sucrose over water (the ratio of sucrose to total fluid intake) was calculated. The rat had free access to food before and during the experiment and the food intake was measured during the three hours of sucrose consumption. Sucrose was diluted in tap water to a final concentration of 5% (w/v). The data were analyzed by a paired t-test analysis.

## Results

### Genetics of the ghrelin signalling system and sucrose intake in a population cohort

None of the SNPs differed significantly from HWE. SNP and haplotype frequencies were similar to those previously reported [26], [27] and did not differ significantly between high- and low alcohol consumers (data not shown).

When analyzing sucrose intake and the above mentioned genetic markers in *GHRL* and *GHSR* in the population cohort, associations with haplotypes (AGACGT and GACGT) in the *GHRL* and increased sucrose consumption were found (Table 2), i.e. carriers of these haplotypes have an increased sucrose consumption compared to non-carriers. More specifically, the haplotype GACGT was associated with increased sucrose intake when analyzing all individuals (p = 0.037) and high alcohol consumers only (p = 0.047) while a trend for the same association was found in high consuming males (p = 0.065). However, these association did not hold for multiple testing (p_c_ = 0.074, p_c_ = 0.090 and p_c_ = 0.125, respectively). The nearly identical haplotype, except for an additional SNP in the beginning, AGACGT was associated with sucrose intake in all males (p_c_ = 0.045). Although these association are quite modest, the largest representing a mere 20 g sucrose/day per allele which corresponds roughly to 7 pieces of sugar or about one can of soda, they still implicate a relationship between GHRL genetics, sucrose and alcohol consumption, specifically in males.

**Table 2:** Haplotypes of the GHRL are associated with high sucrose intake.

Gene	SNPs	Haplo-type	Group	n =	Effect ing/allele (±SE)	p-value	p_c_-value
*GHRL*	2–6	-GACGT	High and Low EtOH	571	7 (±4)	0.037	0.074
	2–6	-GACGT	High EtOH	279	10 (±5)	0.047	0.090
	1–6	AGACGT	High and Low EtOH males	344	20 (±10)	0.044	0.045
	2–6	-GACGT	High EtOH males	248	10 (±6)	0.065	0.125

*SNPs = single nucleotide polymorphism; GHRL = pro-ghrelin gene; SE = standard error; p_c_-values are calculated using regression models including associated haplotypes and relevant covariates and are presented as values corrected for multiple testing by permutation analysis; p-values are based on likelihood ratios test while the 95% CIs are calculated using Wald statistics.*

### The GHS-R1A antagonist decreases the sucrose intake in the intermittent access 5% sucrose two-bottle-choice drinking paradigm

**Figure 1:**
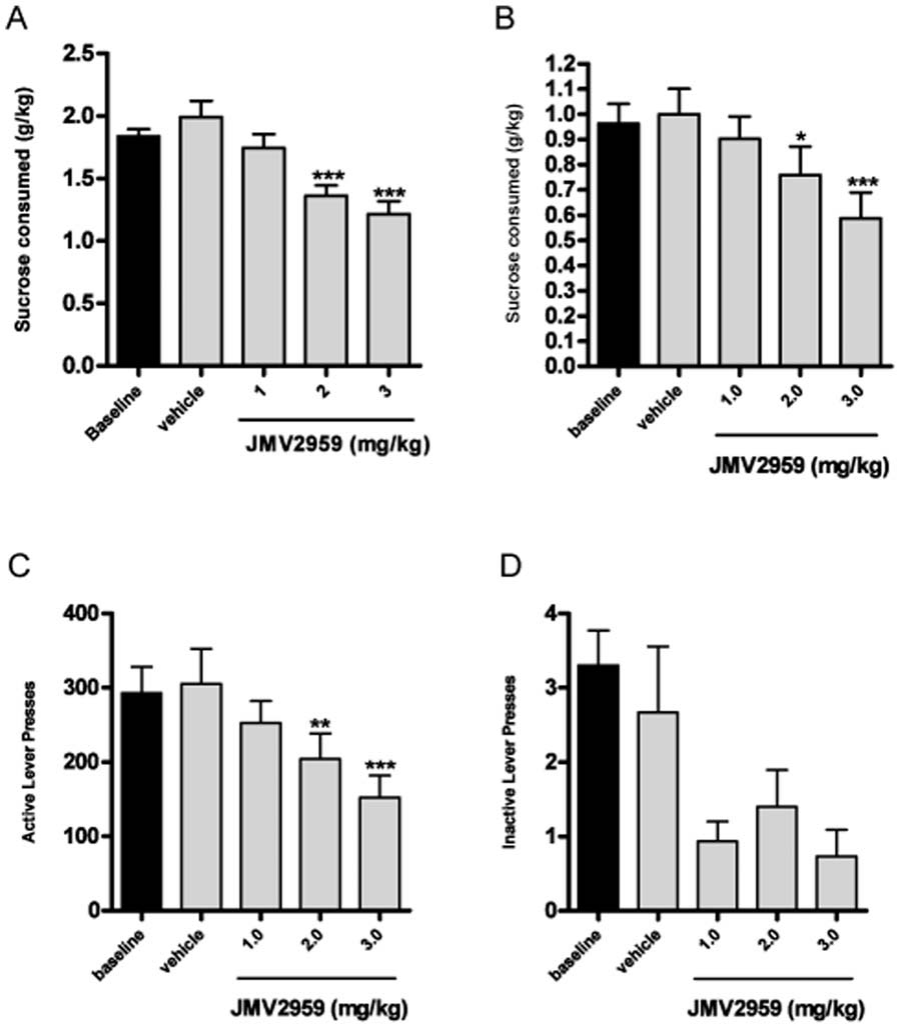
The GHS-R1A antagonist reduces voluntary sucrose- intake and operant self-administration in rats. (A) JMV2959 treatment (2 and 3 mg/kg) decreased the sucrose consumption (g/kg) in the intermittent access 5% sucrose two-bottle-choice drinking paradigm. (B) GHS-R1A antagonist treatment (2 and 3 mg/kg) reduced the consumption of 5% sucrose (g/kg) in the operant self-administration chamber, (C) JMV2959 (2 and 3 mg/kg) significantly decreased the number of presses on the active lever compared to vehicle, (D) but did not affect the number of presses on the inactive lever.

We examined the effect of JMV2959 on voluntary sucrose consumption in heavy drinking rats. Once the rats had maintained stable baseline sucrose consumption for 7 weeks (∼20 sessions), JMV2959 (1, 2, and 3 mg/kg, i.p.) was administered 20 min before the presentation of the sucrose bottles. We found that JMV2959 treatment had an overall main effect on sucrose consumption (g/kg) at the 30 min time point [30 min: F(3,11) = 15.03, P<0.0001, n = 12], but showed no effect at the 6 hr and 24 hr time points [6 hr: F(3,11) = 1.10, n.s; 24 hr: F(3,11) = 1.06, n.s]. Post hoc analysis revealed a significant effect of JMV2959 (2 and 3 mg/kg) at the 30 min time point (Figure 1A). There was also an overall main effect of JMV2959 on the preference for sucrose over water at the 30 min time point [30 min: F(3,11) = 4.11, P<0.05](data not shown), but showed no effect at the 6 hr and 24 hr time points [6 hr: F(3,11) = 0.54, n.s ; 24 hr: F(3,11) = 0.69, n.s](data not shown). JMV2959 had no overall main effect on water consumption at any of the time points in the sucrose consuming rats [30 min: F(3,11) = 0.74, n.s; 6 hr: F(3,11) = 0.12, n.s ; 24 hr: F(3,11) = 0.94, n.s](data not shown). JMV2959 treatment had no overall main effect on body weight [F(3, 11) = 1.29, n.s.](data not shown).

### The GHS-R1A antagonist reduces sucrose consumption in the operant self-administration procedures

We examined the effect of JMV2959 on sucrose self-administration in Long-Evans rats. JMV2959 (1, 2, and 3 mg/kg, i.p.) was administered 20 min before the operant session, once the rats had maintained a stable level of responding for 5 weeks (∼25 FR3 sessions). JMV2959 treatment had an overall main effect on 5% sucrose self-administration [F(3,14) = 10.00, n = 15] (Figure 1B). Post hoc analysis revealed that both the 2 and 3 mg/kg doses of JMV2959 (mg/kg) significantly decreased the number of presses on the active lever compared to vehicle (Figure 1C). Furthermore, JMV2959 had no overall main effect on the number of presses on the inactive lever (Figure 1D) [F(3,14) = 2.81, n.s]. There was no significant effect of JMV2959 treatment on food intake in the sucrose trained animals [F(3,14) = 1.42, n.s](data not shown). JMV2959 treatment had no overall main effect on body weight [F(3,14) = 2.75, n.s](data not shown).

### The GHS-R1A antagonist decreases the saccharin intake in the saccharin consumption paradigm

**Figure 2:**
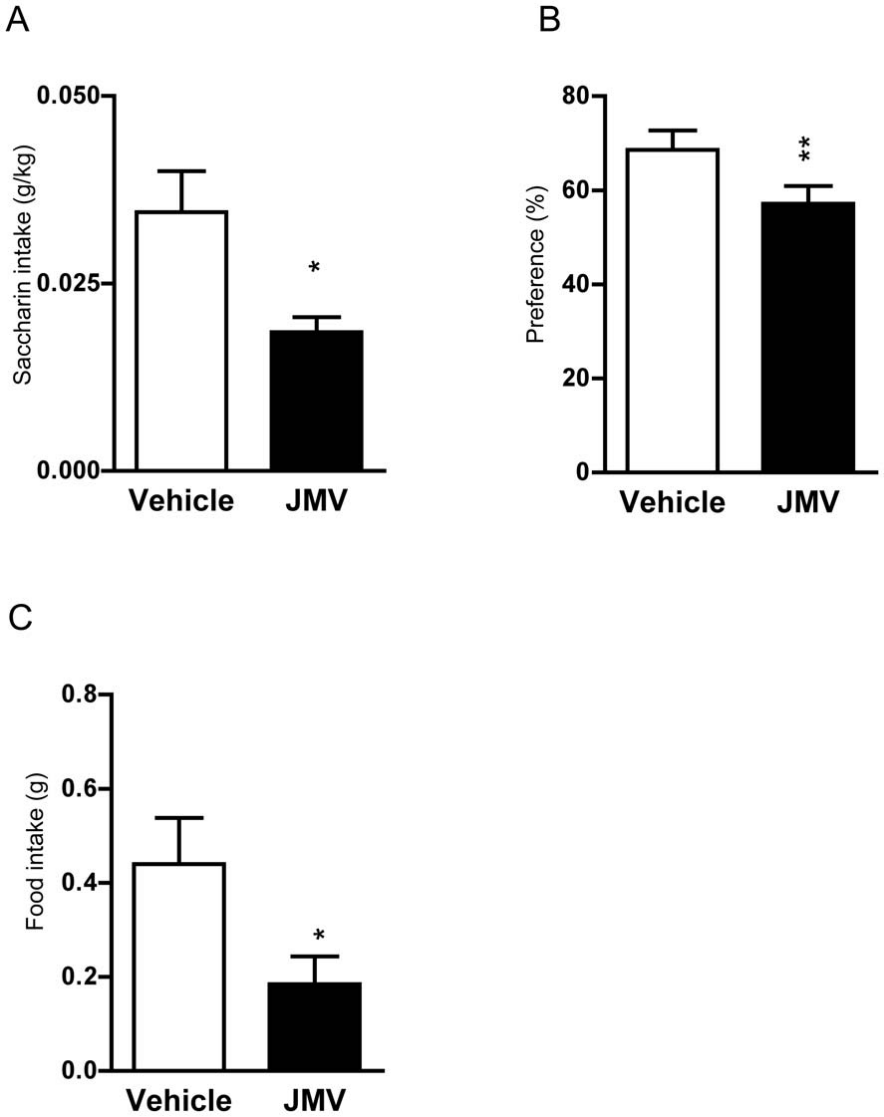
The GHS-R1A antagonist decreases saccharin intake in mice. (A) The GHS-R1A antagonist (6 mg/kg) decreased the saccharin intake (g/kg) during the three hour limited access paradigm in mice compared to vehicle treatment. (B) JMV2959 (6 mg/kg) decreased the saccharin preference over water (%). (C) The food intake (g) over the three hour period was decreased compared to vehicle treatment.

In mice, we examined the effect of JMV2959 on voluntary saccharin consumption. Mice were trained to consume saccharin and established stable baseline consumption. At baseline, there was no difference in saccharin intake between mice later subjected to different treatments (vehicle 0.04±0.002 g/kg/3 hrs; JMV2959 0.05±0.002 g/kg/3 hrs) [F(1,23) = 0.023, p = 0.881]. In addition, no difference in water intake was observed (vehicle 0.24±0.01 g/3 hrs; JMV2959 0.30±0.20 g/3 hrs) [F(1,23) = 1.63, p = 0.213] or 24 hour food intake (vehicle 0.76±0.03 g; JMV2959 0.76±0.04 g [F(1,23) = 0.006, p = 0.939] at baseline (data not shown). Thereafter, JMV2959 (6 mg/kg, i.p.) or vehicle was administered 20 min before the presentation of the saccharin bottle. JMV2959 or vehicle was administered for two subsequent days, and as no difference in saccharin intake or preference was observed between the two treatment days, the graph only shows the average intake. We found that JMV2959 treatment had an overall main effect on saccharin consumption (g/kg) [F(1,23) = 6.52, p = 0.018; n = 12 for JMV2959 and n = 13 for vehicle treatment] (Figure 2A) as well as on saccharin preference (%) [F(1,23) = 8.67, p = 0.007] (Figure 2B). No effect on water intake (g) was observed following JMV2959 treatment [F(1,23) = 1.32, p = 0.261](data not shown). Additionally, we found that JMV2959 treatment decreased the 90 min food consumption (g) [F(1,23) = 7.57, p = 0.011] (Figure 2C), but not the 24 hour food intake [F(1,23) = 0.229, p = 0.637](data not shown). After treatment the mice were untreated for two days and there was no difference in saccharin intake between mice subjected to different treatments (vehicle 0.05±0.005 g/kg/3 hrs; JMV2959 0.05±0.003 g/kg/3 hrs) [F(1,23) = 0.533, p = 0.473]. In addition, no difference in water intake was observed (vehicle 0.30±0.02 g/90 min; JMV2959 0.32±0.02 g/90 min) [F(1,23) = 0.528, p = 0.476] or 24 hour food intake (vehicle 0.81±0.07 g; JMV2959 1.00±0.07 g) [F(1,23) = 2.413, p = 0.134] after treatment (data not shown).

### The GHS-R1A antagonist does not affect saccharin consumption in the taste aversion set-up

**Figure 3:**
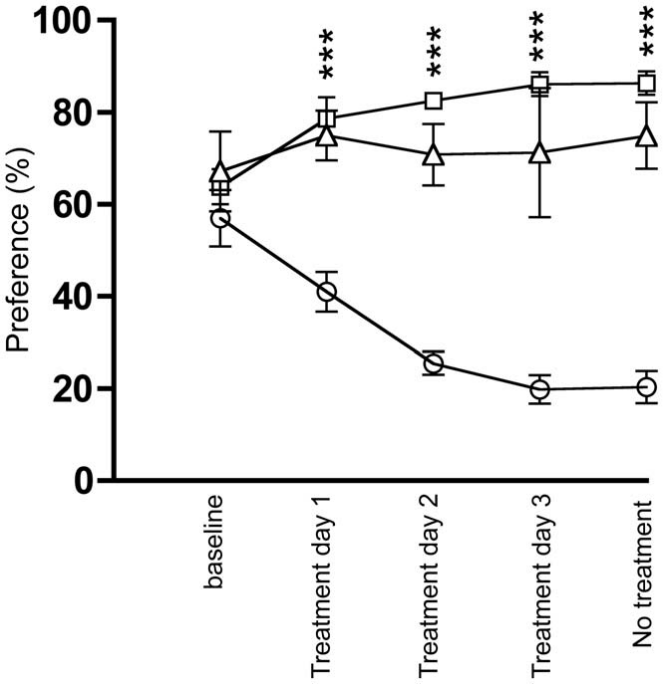
The GHS-R1A antagonist does not induce a taste aversion in mice. The GHS-R1A antagonist (6 mg/kg) did not affect the preference for saccharin over water compared to vehicle treatment in the taste aversion experiment. LiCl, on the other hand, decreased the preference for saccharin over water compared to both JMV2959 and vehicle treatment (Square  =  vehicle; triangle  =  JMV2959; Circle  =  LiCl).

The effects of JMV2959 or LiCl on voluntary saccharin consumption in a taste aversion experiment in mice were studied. At baseline, i.e. the first saccharin consumption day where mice were treated after saccharin exposure, no differences in saccharin intake [F(2,12) = 0.827, p = 0.461, n = 5], saccharin preference (F(2,12) = 0.633, p = 0.548), water intake [F(2,12) = 0.395, p = 0.682] (Figure 3) or food intake [F(2,12) = 0.907, p = 0.430](data not shown) were observed.

We found that treatment had an overall main effect on saccharin consumption (g/kg) [F(2,12) = 16.865, p = 0.003, n = 5] as well as on saccharin preference (%) [F(2,12) = 34.92, p<0.001]. Post hoc test showed that this was due to that LiCl reduced the saccharin intake as well as preference each day compared to both vehicle (p = 0.0001; p<0.0001 respectively) and JMV2959 (p = 0.0021; p<0.0001 respectively). There was no difference in saccharin intake or preference between vehicle and JMV2959 (p = 0.102; p = 0.164 respectively). Moreover, treatment had an overall main effect on water consumption (g) [F(2,12) = 18.21, p = 0.002] (Figure 3). Post hoc test showed that this was due to a compensatory increase in water intake after LiCl treatment compared to both vehicle (p = 0.0001) and JMV2959 (p = 0.0005) treatment. There was no difference in water intake between vehicle and JMV2959 (p = 0.372) treatment. Moreover, treatment had no overall main effect on food consumption (g) [F(2,12) = 2.61, p = 0.115](data not shown).

The last day, when the mice were untreated, we found an overall main effect of previous treatment on saccharin consumption (g/kg) [F(2,12) = 15.092, p = 0.0005] as well as on saccharin preference (%) [F(2,12) = 34.92, p<0.001]. Post hoc test showed that this was due to the previous LiCl treatment, which reduced the saccharin intake as well as preference compared to previous vehicle (p = 0.0001; p<0.0001 respectively) and JMV2959 (p = 0.0072; p<0.0001 respectively) treatment (Figure 3). There was no difference in saccharin intake or preference between previous vehicle and JMV2959 (p = 0.0451; p = 0.124 respectively) treatment. Moreover, previous treatment had an overall main effect on water consumption (g) [F(2,12) = 20.201, p = 0.002](data not shown). Post hoc test showed that this was due to a compensatory increase in water intake after previous LiCl treatment compared to both vehicle (p = 0.0001) and JMV2959 (p = 0.0002) treatment. There was no difference in water intake between previous vehicle and JMV2959 (p = 0.850) treatment. Moreover, previous treatment had no overall main effect on food consumption (g) [F(2,12) = 2.14, p = 0.161](data not shown).

### Ghrelin increases the sucrose intake in the sucrose consumption paradigm

**Figure 4:**
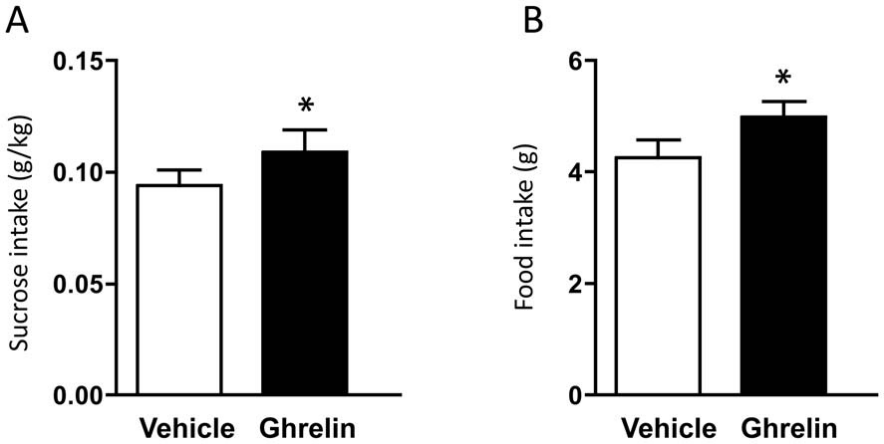
Peripheral ghrelin administration increases the sucrose intake in rats. Peripheral ghrelin (0.33 mg/kg) administration compared to vehicle treatment increases (A) the sucrose intake (g/kg) as well as (B) food intake (g) in the three hour 5% sucrose two-bottle-choice drinking paradigm in rats.

In rats, we examined the effect of peripheral administration of ghrelin on voluntary sucrose consumption. Rats were trained to consume sucrose and established stable baseline consumption (sucrose intake 0.11±0.01 g/kg/ 3 hrs; sucrose preference 96.50±0.51%; water intake 0.86±0.08 g/3 hrs; total fluid intake 28.01±1.98 g/3 hrs; food intake 4.91±0.26 g/3 hrs). Thereafter, ghrelin (0.33 mg/kg, i.p.) or vehicle was administered 10 min before the presentation of the sucrose bottle. We found that ghrelin treatment increased the sucrose consumption in rats (g/kg) (p = 0.0273) (Figure 4A) as well as the food intake (p = 0.0479) (Figure 4B) in rats. No effect on sucrose preference (vehicle 94.44±0.79%; ghrelin 94.87±0.61%), water intake (vehicle 1.56±0.28 g; ghrelin 1.46±0.19 g) or total fluid intake (vehicle 28.39±2.15 g; ghrelin 31.66±3.24 g) was observed following ghrelin treatment (p = 0.663, p = 0.786, 0.119 respectively).

## Discussion

In the present study we found associations with the haplotype AGACGT, and its shorter version, GACGT, in the *GHRL* and increased sucrose consumption. The haplotype GACGT was associated with increased sucrose intake in all individuals included in the study, and a trend for the same association was specifically seen in the high alcohol consumers. The nearly identical haplotype, AGACGT, in the *GHRL* was associated with high sucrose intake in male subjects. The preclinical data show that the GHS-R1A antagonist reduces the intake of sucrose as well as saccharin in rodents, as well as decreases self-administration of sucrose in rats. Moreover, we found that the GHS-R1A antagonist did not induce a taste aversion. Finally, the present study shows that peripheral ghrelin administration increases the intake of sucrose in rats. Taken together, this suggests that ghrelin signalling, including the GHS-R1A, is important for regulation of the consumption of rewarding substances in general, and that this is independent of both the caloric content and any aversive effect.

In the genetic part of this study, an association between a *GHRL* haplotype and higher sucrose intake, particularly in males, was found. This is in line with previous studies where male gender specifically predicts a high preference for sweets [14], [39], and that sweet preference is a highly genetically determined trait [15]. Even though the effect size of this association was quite modest and the functional significance of this haplotype is yet unknown, it may be suggested that the associated ghrelin-encoding polymorphisms might influence the ability of the ghrelin signalling system to regulate the intake of rewarding substances. Supportively, in the preclinical experiments, we showed that pharmacological suppression of the GHS-R1A reduced high sucrose consumption as well as operant self-administration of sucrose in rats. Further, peripheral administration of ghrelin increased the intake and preference of sucrose in rats. Supportively, it was recently shown that ghrelin (administered centrally as well as peripherally) increases whereas GHS-R1A antagonism suppresses the motivation to consume sucrose [37] and that central grelin administration increase the intake of sucrose in rats [38]. Given that the orexigenic peptide, ghrelin, regulates energy balance [8], the reduced sucrose intake may be related to its caloric content. However, this appears less likely since we here show that GHS-R1A antagonism decreases consumption of saccharin, a non-caloric sweetener, in mice. Recently, peripheral ghrelin administration was shown to increase the consumption of saccharin in wild type but not GHS-R1A knockout mice [40]. Finally, we found that JMV2959 did not induce a taste aversion in mice, as LiCl does, suggesting that the reduced intake of sweet substances is due to suppressed reward mechanisms rather than aversion.

A trend for an association between the *GHRL* haplotype, AGACGT, and higher sucrose intake was also seen specifically for the group with higher alcohol consumption. As this study investigated a sample of individuals from a population cohort, this trend needs to be further investigated in an alcohol dependent population with a higher alcohol intake and possible co-morbid eating disorder. These data are, however, of interest since previously published studies show a positive correlation with the response to sweet taste and excessive alcohol intake in humans [18], [41], and that SNPs and haplotypes of both the *GHRL* and *GHSR* have been associated with increased weight in alcohol dependent individuals [26]. Moreover, animals bred for high saccharin preference show increased alcohol consumption compared to low saccharin-preferring animals [42]. Given that human imaging studies also reveal an underlying disruption in the reward systems in addictive behaviors, including alcohol use disorder and binge eating [1], [2], [3], we suggest that underlying neurobiological mechanisms for such addictive behaviors include central ghrelin signalling at the level of the reward systems, such as the cholinergic-dopaminergic reward link [43].

From both preclinical and clinical data it now seems clear that central ghrelin signalling, including both the peptide ghrelin and its receptor, have a role in reward regulation. Indeed, here we showed that ghrelin increases whereas GHS-R1A antagonist reduced the intake and self-administration of sucrose in fed rats. Additionally, recent studies show that ghrelin increases the intake of sucrose in rats fed *ad libitum* as well as in rats subjected to mild food restriction [37], [38]. Furthermore, cocaine-seeking is associated with elevated plasma levels of ghrelin and a peripheral ghrelin injection enhances cocaine-induced locomotor stimulation and conditioned place preference [44], [45], [46]. Moreover, ghrelin administration in to the brain ventricles or into the VTA or LDTg, important reward nodes, increases alcohol consumption in mice [5] and the alcohol-induced locomotor stimulation and accumbal dopamine releases is attenuated in ghrelin knockout mice [4]. Further, ghrelin increases foraging in rats [47] and enhances the activity of reward-related brain nodes in humans subjected to food-related cues [48]. In human genetic studies a *GHRL* haplotype has been associated with paternal heredity of alcohol-use disorder [27], of increased weight in alcohol dependent individuals [26], as well as with increased sucrose consumption, as shown in the present study. Regarding the GHS-R1A, it was recently shown that GHS-R1A suppression reduces the intake of as well as the motivation to obtain palatable food [6] and attenuates alcohol-, cocaine-, and amphetamine-induced reward, as measured by locomotor activity, accumbal dopamine release and conditioned place preference [5], [13]. Further central or peripheral administration of a GHS-R1A antagonist reduces alcohol consumption as well as self-administration of alcohol in both mice and rats [5], [34], [49]. Supportively, present and recent studies have shown that GHS-R1A antagonism decreases the intake of sweet substances in rodents [37], [40]. Associations with one SNP and of haplotypes in *GHSR* and alcohol consumption, increased weight in alcohol dependent individuals, as well as with smoking in female alcohol dependence have been shown [26], [27]. Conclusively, these clinical and preclinical findings suggest that ghrelin signalling may regulate both the intake of and search for rewarding substances, and a possible role for ghrelin signalling in patients with multiple addictions.

It now seems clear that ghrelin activates the cholinergic-dopaminergic reward link and that ghrelin thereby increase the incentive value for motivated behaviors i.e. reward-seeking [9], [10]. Given that the present and recent studies show that ghrelin increase and that the GHS-R1A antagonist reduces the intake of sucrose in rats [37], [38], it should be considered that the role of ghrelin signaling for the consumption of sweets is related to the endogenous peptide ghrelin and/or to the GHS-R1A. However, the specific mechanisms through which ghrelin signalling may regulate the intake of rewarding substances need to be further elucidated. One possibility is that ghrelin amplifies dopamine signalling in the mesolimbic dopamine system through cross-talk involving heterodimerization of GHS-R1A and dopamine D_1_-like receptors, receptors co-localized on dopaminergic neurons in the VTA [50]. Another possibility is that the GHS-R1A via its constitutive activity regulates the sensitivity of the mesolimbic dopamine system, and the ability of drugs of abuse to activate this system [51].

Here, we showed that haplotypes in the *GHRL* are associated with increased sucrose consumption in humans. We also showed that a GHS-R1A antagonist reduces the consumption of both sucrose and saccharin, as well as the self-administration of sucrose in rodents. Collectively, our data provide a clear indication that the central ghrelin signalling system, via GHS-R1A, is involved in regulating the intake of rewarding substance and hence, provides a novel target for the development of drug strategies to treat addictive behaviors.
