# Correction: The Ghrelin Signalling System Is Involved in the Consumption of Sweets

**DOI:** 10.1371/annotation/69c18638-12de-4f08-9a3c-f8c7bf3a0cc6

**Published:** 2014-01-03

**Authors:** Sara Landgren, Jeffrey A. Simms, Dag S. Thelle, Elisabeth Strandhagen, Selena E. Bartlett, Jörgen A. Engel, Elisabet Jerlhag

The authors wish to make the following correction:

In our article, we declared that the study was partly funded by the Swedish Council for Tobacco Research (Rådet för Medicinsk Tobaksforskning). While the authors have received a grant from the Swedish Council for Tobacco Research, this was for a different project. The current study was therefore not funded by the Swedish Council for Tobacco Research and the declaration of funding should not have included a reference to this body. 

